# A novel idiopathic transient tubulopathy associated with exercise-induced-seizures: A case report

**DOI:** 10.1016/j.ebr.2026.100859

**Published:** 2026-03-11

**Authors:** Héctor M. Ramos-Zaldívar, Eduardo Smelin Perdomo Domínguez, Joselin Michelle Monterroso-Reyes, Astrid Yohaly Rivera Caballero, Julie R. Jones, Edison Salas-Huenuleo, María Fernanda Hernández Villarroel, Franz Villarroel-Espíndola, Sashenka Silva-Jiménez, Rodrigo E. Santa Cruz-Flores, Cristobal Andrés Padilla Jeria, Catalina Raquel González Urriola, Martín P. Fierro

**Affiliations:** a Laboratorio de Medicina Traslacional, Centro de Investigación e Innovación en Cáncer, Instituto Oncológico Fundación Arturo López Pérez, Santiago, Chile; b Sapiens Wisdom Research, Santiago, Chile; c GIMUNICAH, Faculty of Medicine, Pontificia Universidad Católica de Honduras, San Pedro Sula, Honduras; d Greenwood Genetic Center, Greenwood, SC, USA; e Advanced Integrated Technologies (AINTECH), Santiago, Chile; f Faculty of Medicine, Universidad de Cuenca, Cuenca, Ecuador; g Academia de Desarrollo de Talentos UC Rolando Chuaqui, Pontificia Universidad Católica de Chile, Santiago, Chile; h Universidad de Concepción, Santiago, Chile; i Ciencias de la Salud, Universidad Mayor, Santiago, Chile

**Keywords:** Seizures, Exercise, Renal tubulopathy, Idiopathic, Epilepsy

## Abstract

Although exercise is generally considered beneficial for individuals with epilepsy, rare cases of exercise-induced seizures have been reported. Physical activity influences renal hemodynamics and electrolyte handling, both essential for neurological stability. Disruptions in renal compensatory mechanisms may contribute to seizure susceptibility during exertion. We describe a 26-year-old male patient with exercise-induced seizures over a period of four months, occurring during high-intensity activity, preceded by visual aura and followed by tonic-clonic episodes. Laboratory workup revealed persistent hypophosphatemia (nadir 1.4 mg/dL) and hyperuricemia (up to 15.8 mg/dL). Brain imaging and EEG were unremarkable, and extensive metabolic, autoimmune, toxicological, and genetic investigations ruled out structural, infectious, and hereditary epileptic causes. Spot urine analysis, obtained in the absence of recent exercise, revealed markedly elevated urinary losses of sodium (UNa 207.1 mEq/L), potassium (UK > 100 mEq/L), and chloride (UCl 281.8 mEq/L) suggesting a renal tubular defect. The patient’s condition stabilized with antiseizure medication and electrolyte supplementation therapy, and he remained seizure-free for five years following normalization of serum phosphate and uric acid levels. The absence of identifiable structural or genetic pathology, combined with spontaneous clinical resolution, suggests a reversible functional disorder. In patients with normal neurological and cardiac evaluations, renal electrolyte disturbances should be considered in the differential diagnosis of exercise-related seizures. Spot urinary electrolyte analysis can be a valuable diagnostic tool in atypical cases, particularly when neuroimaging and interictal findings are unremarkable.

## Highlights

• First case of exercise-induced seizures linked to transient renal tubulopathy.
• Marked urinary sodium, potassium, and chloride loss without structural abnormality.
• Genetic, autoimmune, and toxic causes excluded by comprehensive analyses.
• Full clinical recovery after normalization of phosphate and uric acid levels.
• Spot urinary electrolytes recommended in unexplained exertion-related seizures.

## 1 Introduction

The role of physical exercise as a factor in triggering seizures has been extensively debated. People with epilepsy have been discouraged from participating in physical activities or sports due to fear, overprotection, and unawareness about the potential benefits of these activities. [1] Physical exercise or participating in sports has been shown to improve seizure control and provide psychosocial benefits for people with epilepsy. [2] In 2016, the International League Against Epilepsy (ILAE) Task Force on Sports and Epilepsy reported that physical exercise and/or sport activities are unlikely to induce seizures. [3] Accordingly, contemporary evidence supports physical exercise as safe and beneficial for most individuals with epilepsy, with exercise-induced seizures being rare and typically associated with focal epileptic syndromes or structural abnormalities, rather than metabolic causes. [4], [5] However, there have been reports of exercise-induced seizures occurring during activities such as running, jogging, stair climbing, and various sports; nonetheless, the underlying mechanisms remain largely unclear. [6], [7], [8], [9] Fatigue, stress, hyperventilation, and ionic/metabolic disturbances have been described as possible precipitating factors for exercise-induced seizures, but no link has been established between these factors and seizure frequency. [10]

Renal function is important for maintaining homeostasis and reducing electrolyte excretion during exercise. [11] There are reports that the rates of sodium, chloride, calcium, and phosphate excretion decrease after exercise. [11] Most studies report that potassium excretion is not consistently affected by moderate to heavy exercise. [11] The mechanism behind this phenomenon is related to increased tubular reabsorption and any disturbances in this compensatory mechanism could trigger brain electrolyte derangements. This could explain the unknown underlying mechanism of exercise-induced seizures.

We report a case with a history of exercise-induced seizures associated with significant urinary electrolyte loss. This case broadens the differential diagnosis of exercise-induced seizures by illustrating the role of renal electrolyte imbalance in seizure generation. It emphasizes the need for a comprehensive metabolic and renal evaluation in patients presenting with exertion-related seizures of unclear etiology.

## 2 Case report

We report the case of a 26-year-old male patient with a history of two episodes of seizures occurring exclusively during high-intensity exercise over a four-month period. He was born to non-consanguineous parents, with no relevant prenatal history and no known family history of epilepsy or other diseases. His past medical history was significant for allergic rhinitis, occasional cannabis consumption, and multiple surgeries, including septoplasty, turbinectomy, adenoidectomy, inguinal hernia repair, and bilateral gynecomastia surgery. He had a height of 1.70 m, weighed 72 kg, and had a body mass index (BMI) of approximately 24.9 kg/m^2^.

During the preoperative evaluation for gynecomastia surgery (four years before the first seizure), hyperuricemia (7.2 mg/dL; laboratory reference range 3.4–7.0 mg/dL) and hypophosphatemia (2.4 mg/dL; laboratory reference range 2.5–4.5 mg/dL) were present. Thyroid function tests were normal. Follicle-stimulating hormone (FSH), luteinizing hormone (LH), beta-human chorionic gonadotropin (beta-hCG), alpha-fetoprotein (AFP), testosterone, and prolactin levels were within normal ranges. A complete blood count (CBC) was normal. Scrotal ultrasound revealed mild distention of the left pampiniform venous plexus. Histopathological evaluation of gynecomastia tissue specimens was consistent with a fibrous pattern. The patient also reported chronic therapy with sertraline (50 mg once daily) and clonazepam (0.5 mg every 24 h), initiated within the year preceding his first seizure episode.

**Fig. 1:**
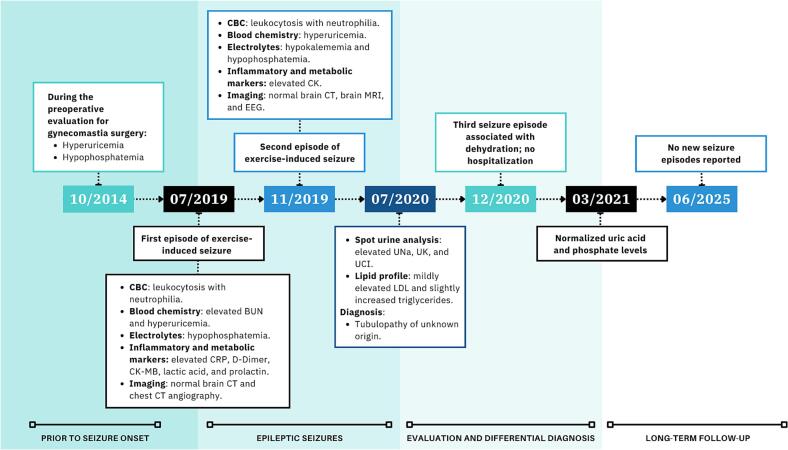
Clinical timeline of exercise-induced seizures and associated metabolic abnormalities.

The first episode occurred while performing an intense boxing workout (Fig. 1). The patient reported using Lipo 6 Black Ultra (All Nutrition) as a metabolic accelerator and pre-workout supplement. According to the manufacturer, this product contains caffeine anhydrous and is delivered in a vegetable capsule composed of hypromellose and chlorophyllin. Additional ingredients include microcrystalline cellulose, guarana extract (Paullinia cupana, standardized to 22% caffeine), magnesium stearate, cocoa extract (*Theobroma cacao*, containing 0.25% caffeine), and silicon dioxide. It also provides chromium (as chromium picolinate) and vitamin B12 (as cyanocobalamin).

During exercise he reported phosphenes and brief visual hallucinations seconds before losing consciousness. Witnesses described short tonic seizures affecting the abdomen and arms in extension (15 s), followed by tonic-clonic seizures lasting approximately one minute, with recovery of consciousness five minutes later. Upon hospitalization, the patient was afebrile and normotensive, with no evidence of cardiac or respiratory abnormalities, and no motor or sensory deficits.

Routine laboratory analyses, including complete blood count (CBC), showed leukocytosis (14000/mm^3^) with neutrophilia (11760/mm^3^). Blood chemistry tests revealed slightly elevated blood urea nitrogen (21 mg/dL) and hyperuricemia (15.8 mg/dL). Venous gasometry, blood coagulation tests, and vitamin B12 were within normal limits. Liver function tests, including aspartate transaminase (AST), alanine transaminase (ALT), total bilirubin, direct bilirubin, indirect bilirubin, alkaline phosphatase (ALP), and gamma-glutamyl transferase (GGT), were within normal ranges. Serum electrolyte assessment revealed hypophosphatemia (2.4 mg/dL). Serum sodium, potassium, chloride, calcium, and magnesium levels were normal. Creatinine (1.00 mg/dL) and glomerular filtration rate (97 mL/min/1.73 m^2^) were within normal range.

Additional laboratory analyses showed nonspecific elevated C-reactive protein (CRP) (17.6 mg/L); elevated D-dimer (284.23 ng/mL); slightly elevated creatine kinase-myocardial band (CK-MB) (29 U/L); total creatine kinase (CK) value of 424 U/L, which increased the following day to 1094 U/L before decreasing 72 h later to 897 U/L; increased lactic acid level of 23.10 mg/dL; and elevated prolactin (25.2 ng/mL). Thyroid function tests were within normal ranges. Viral screens for respiratory syncytial virus (RSV), influenza A and B virus, and adenovirus were negative. Venereal Disease Research Laboratory (VDRL) and Rapid Plasma Reagin (RPR) tests were non-reactive as well.

Computed tomography (CT) of the brain and CT angiography of the chest showed no abnormalities. The patient was discharged on levetiracetam 500 mg orally once daily for maintenance.

The second episode of exercise-induced seizure occurred four months later (Fig. 1). No metabolic accelerator or pre-workout supplement was used on this occasion, arguing against a causal role of supplementation in seizure occurrence. While performing high-intensity interval training (HIIT), a form of exercise consisting of repeated bouts of vigorous aerobic activity (65–90% of VO_2_peak or 75–95% of peak heart rate) interspersed with short periods of active or passive recovery, [12], [13] the patient developed a visual aura (white/vivid-colored lights) with subsequent loss of consciousness. Witnesses stated that he was able to call for help and lie down on the ground before developing a tonic-clonic seizure. He bit the lateral aspect of his tongue, but no sphincter dysfunction was present.

A CBC was remarkable for leukocytosis (17400/mm^3^) with neutrophilia (15121/mm^3^). The biochemical profile revealed hyperuricemia (13.4 mg/dL), with normal creatinine (1.04 mg/dL) and glomerular filtration rate (>60 mL/min/1.73 m^2^). Liver function tests, including AST, ALT, total bilirubin, ALP, and GGT, were within normal ranges. Routine blood coagulation tests were normal. Serum electrolyte evaluation showed hypokalemia (2.9 mEq/L) and hypophosphatemia (1.4 mg/dL); sodium, chloride, and calcium were within normal ranges.

**Fig. 2:**
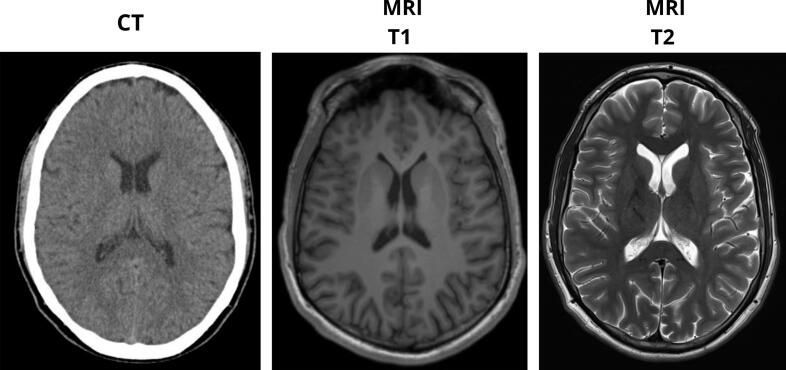
Neuroimaging during the diagnostic workup of exercise-induced seizures. Representative axial slices from brain imaging studies performed after the second seizure episode: non-contrast computed tomography (CT), and magnetic resonance imaging (MRI) T1- and T2-weighted sequences. All neuroimaging results were unremarkable, showing no structural abnormalities.

Regarding neurological assessment, continuous video electroencephalography (EEG) monitoring showed no epileptiform activity. Both contrast-enhanced computed tomography (CT) and magnetic resonance imaging (MRI) of the brain were normal (Fig. 2). A normal electrocardiogram was reported. Additional laboratory analyses showed a normal CRP level and a total CK value of 360 U/L.

The neurology department categorized the clinical presentation as an epilepsy syndrome. The therapeutic approach for seizures consisted of intravenous levetiracetam 1 g twice daily. The patient's hypokalemia was treated with intravenous and oral preparations of potassium chloride (Slow-K 1200 mg TID for 7 days). The patient was discharged against medical advice despite recommendations for further analyses, with a diagnosis of occipital focal epilepsy with secondary generalization associated with moderate hypokalemia. This diagnosis was considered provisional and based on the acute clinical presentation, pending further longitudinal evaluation. At discharge, the patient was prescribed the following outpatient regimen: levetiracetam 1 g every 12 h orally for maintenance, and Slow-K extended-release tablets, 1200 mg every 8 h orally for 7 days. After this event, the patient did not resume high-intensity interval training but continued to engage in light to moderate physical activity without recurrence of seizures.

Given the absence of a clear etiology, we performed an outpatient clinical analysis. Based on the patient’s history of hyperuricemia and hypophosphatemia preceding the seizure episodes, a nephrology consultation was advised for comprehensive renal assessment. Spot urinary electrolytes, obtained in the absence of recent exercise, revealed markedly elevated sodium (UNa 207.1 mEq/L; values > 50 mEq/L suggest impaired renal sodium conservation [14]), potassium (UK > 100 mEq/L; values > 20 mEq/L indicate inappropriate renal potassium loss ^15^), and chloride (UCl 281.8 mEq/L; >20 mEq/L generally suggest elevated renal chloride excretion [15]), while magnesium (6.3 mEq/L), phosphate (52 mg/dL), and glucose (0.07 g/L) were within normal limits. Serum sodium, potassium, chloride, magnesium, and glucose were within normal range. Urinalysis was normal, with no proteinuria. Glomerular filtration rate (assessed using MDRD-4 and CKD-EPI), insulin, aldosterone, renin, and aldosterone/renin ratio were within normal limits. Thyroid function tests were within normal ranges. Lipid profile showed: total cholesterol of 177 mg/dL, high-density lipoprotein (HDL) of 40 mg/dL, mildly increased low-density lipoprotein (LDL) of 106 mg/dL, very low-density lipoprotein (VLDL) value of 31 mg/dL, and slightly increased triglycerides (155 mg/dL).

**Fig. 3:**
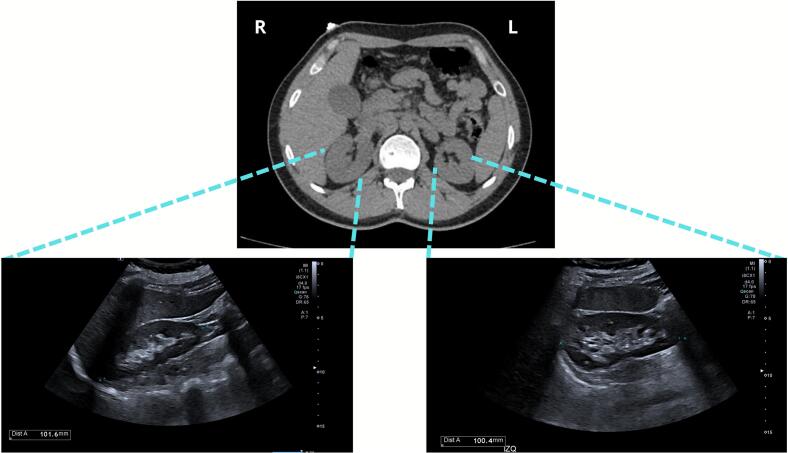
Renal imaging during evaluation for suspected tubulopathy. Abdominal computed tomography (CT) and corresponding renal ultrasound images of the right and left kidneys. Imaging demonstrated normal renal morphology and size (approximately 10 cm longitudinal length), with no evidence of structural abnormalities such as stones, masses, or pelvicalyceal dilation. These findings support the diagnosis of a functional renal tubulopathy in the absence of anatomical defects.

Abdominal ultrasound demonstrated normal hepatobiliary structures (liver, gallbladder, bile ducts), pancreas, spleen, and kidneys (10 cm longitudinal diameter, preserved parenchyma) (Fig. 3), without evidence of stones, masses, pelvicalyceal dilation, or retroperitoneal lymphadenopathy. The abdominal aorta was unremarkable in caliber.

Due to high suspicion of renal tubulopathy, with a Bartter/Gitelman differential diagnosis, a focused exome sequencing panel was performed. The whole exome sequencing data were analyzed specifically to identify alterations in the *SLC12A3* and *CLCNKB* genes. No definitive or likely pathogenic alterations that may be clinically relevant were identified in these genes. Additionally, a QUICK (Quickly Uncovering Important Clinical Knowledge) analysis was performed, and no significant variants related to the patient's clinical features were identified. The QUICK analysis is Greenwood’s NGS-reflex analysis that screens full exome data for pathogenic and likely pathogenic alterations when panel results are negative. The Agilent SureSelect^XT^ Clinical Research Exome V2 kit was used to target known disease-associated exonic regions of the genome (coding sequences and splice junctions of known protein-coding genes associated with disease, as well as an exonic backbone), using genomic DNA from the submitted sample. The targeted regions were sequenced using the Illumina NovaSeq 6000 System with 150 bp paired-end reads. Using Illumina DRAGEN Bio-IT Platform® software, the DNA sequence was aligned and compared to the human genome build 19 (hg19/NCBI build 37). The emedgene® software was used for filtering and analysis of variants.

Screening was performed to rule out tubular damage of autoimmune etiology. This included anti-extractable nuclear antigen (anti-ENA), anti-nuclear antibodies (ANA), anti-DNA antibodies, and rheumatoid factor, all of which were negative. Additionally, C3 and C4 complement levels were normal. Viral hepatitis markers were negative (HBsAg, anti-HBc IgM, and anti-HCV) except for a total anti-HBc, which was determined to be a false positive by two subsequent analyses.

Given the occasional cannabis consumption, heavy metal analysis was performed for arsenic (As), cadmium (Cd), chromium (Cr), copper (Cu), and total lead (Pb) in blood to rule out toxic contamination. The analytical determination was carried out using Inductively Coupled Plasma Optical Emission Spectrometry (ICP-OES PlasmaQuant 9100, Analytik Jena), after chemical digestion of the sample. For this purpose, 1.5 mL aliquots of blood were taken, placed in digestion vessels with an HNO_3_:H_2_O_2_ mixture (4:1), and processed in the Ethos One microwave digester (Milestone) according to the matrix-specific instrumental heating program. Once the sample was digested, the digestion product was transferred to 25 mL flasks and brought to volume with deionized water (Milli-Q® 18.2 MΩ cm). The working solutions and calibration curves were quantified in ICP-OES for As (193.698 nm), Cd (226.502 nm), Cr (267.716 nm), Cu (327.396 nm), Pb (220.353 nm), performed in triplicate, with results were expressed in µg L^−1^ of blood. Analytical characterization was performed considering ten measurements of the blank, while validation of the method’s accuracy was conducted via recovery studies. The method’s precision was evaluated in terms of repeatability and reproducibility by calculating the relative standard deviation (%RSD) of three working solutions prepared at the same known concentration from a standard solution of each metal of 1000 mg L^−1^ (Merck). All heavy metals analyzed, including arsenic, cadmium, chromium, copper, and lead, were within normal limits in blood; copper was the only quantified element (84.18  µg/dL), with levels falling within the expected reference range.

Upon follow-up by our team five years later, the patient reported a third episode occurring one year after the second episode, associated with dehydration during a trip to the beach, without engagement in physical exercise at that time. Once again, the event was preceded by a visual aura (white dots), followed by loss of consciousness and tonic-clonic seizures, with recovery of consciousness 20 min later. The patient was not hospitalized for this dehydration-related episode. Within six months, he resumed physical activities, including running and light gym workouts, without recurrence of seizures. After the event, follow-up tests two years later showed normalization of phosphate (3.1 mg/dL) and uric acid (6.6 mg/dL) levels, and there have been no further reports of seizures over a five-year period. A biochemical profile, including glucose, insulin, hemoglobin A1c test, lactate dehydrogenase, ALP, total cholesterol, total bilirubin, total protein, albumin, AST, ALT, blood coagulation tests, creatinine (1.00 mg/dL), and blood urea nitrogen (17 mg/dL), was unremarkable. Levels of Vitamin D and Vitamin B12 were within normal limits. Thyroid function tests were also normal. A CBC revealed only mild eosinophilia (0.6 × 10^3^/μL), and urinalysis findings were within normal parameters. The neurology department is currently tapering the medication for eventual discontinuation.

## 3 Discussion

We described a patient with a history of exercise-induced seizures associated with significant urinary sodium, potassium, and chloride electrolyte loss. No masses or morphological abnormalities were observed on kidney imaging, and no genetic, autoimmune, or heavy metal-related causes were identified for the functional tubulopathy. Early signs of this transient renal dysregulation, including hyperuricemia and hypophosphatemia, were identified five years before the onset of seizures and resolved two years later, coinciding with the cessation of seizure episodes. Neurological and cardiac evaluations yielded no causative findings.

Although the seizures were temporally associated with metabolic and electrolyte disturbances, antiseizure medication was initiated after the first seizure and later intensified following recurrence, in accordance with standard clinical practice. The clinical course, normal neuroimaging and EEG findings, resolution of seizures following metabolic normalization, and successful tapering of antiseizure medication support the interpretation of a symptomatic metabolic epilepsy rather than a primary epileptic disorder.

In most cases, renal tubular injury is described as a postictal phenomenon. Acute kidney injury has been associated with hyperuricemia and intratubular deposition of uric acid crystals after seizures. [16] Hypophosphatemia has also been proposed as a biomarker temporally associated with epileptic seizures, although it is not considered a trigger. [17] However, in our patient, hyperuricemia and hypophosphatemia were documented several years prior to the first seizure, suggesting an alternative or reverse pathophysiological sequence. Histological analysis would have been useful to confirm these changes. However, given the normal renal imaging, absence of progressive disease, spontaneous clinical improvement, and non-confirmatory genetic and immunologic findings, renal biopsy was not pursued, as it was unlikely to alter management.

Events of hypertension associated with renal tubulopathies have also been linked to seizures, as seen in posterior reversible encephalopathy syndrome (PRES) and familial hyperkalemic hypertension (FHHt). [18], [19] Nevertheless, this mechanism would not account for the manifestations in our patient, as he was normotensive in every evaluation, and no lesions suggestive of PRES were observed on MRI. The onset of symptoms occurred in adulthood, was transient, and there was no family history suggestive of FHHt. The transitory presentation and the lack of sensorineural deafness or ataxia ruled out other tubulopathies related to seizures, such as EAST/SeSAME syndrome caused by channelopathies. [20]

To the best of our knowledge, following a review of the medical literature, there are no case reports describing transient seizures with onset in adulthood that are induced by exercise and caused by a renal tubulopathy. Nevertheless, the concurrent marked loss of sodium, potassium, and chloride in the urine of our patient suggests pathophysiological damage of the apical sodium–potassium-chloride cotransporter (NKCC) in the thick ascending limb of the loop of Henle. [21] Again, the transitory nature of this clinical presentation points away from a genetic cause and more toward infectious or toxic etiologies. Of note, even though it was not analyzed in this clinical case, human cytomegalovirus (HCMV) has previously been reported to significantly decrease NKCC function and protein expression. [22] Loop diuretics such as bumetanide and furosemide are well-established inhibitors of NKCC, but there was no history of their use by our patient. [23] Venoms from lizards, platypuses, and certain scorpions have been shown to indirectly affect NKCC activity. However, there was no history of exposure to these toxins in this case.

The renal tubular injury also suggests an underlying brain electrolyte imbalance that triggered seizures during high-intensity exercise. Exercise is unlikely to represent the primary cause of the renal tubulopathy; rather, high-intensity exertion appears to have acted as a physiological stressor that unmasked a transient, underlying tubular dysfunction, leading to pathological electrolyte loss and seizure susceptibility. Notably, seizures were temporally associated only with high-intensity exercise, whereas light and moderate physical activity were well tolerated, suggesting that pathological electrolyte loss and insufficient renal compensation occurred only under conditions of maximal physiological stress. Few case reports of seizures limited to exercise have been published, and the underlying mechanism is not well elucidated. An underlying occipital epilepsy was considered unlikely given normal neuroimaging and EEG findings, absence of seizures at rest or during light-to-moderate exercise, and sustained seizure remission after metabolic normalization.

Werz reported a case of a 21-year-old woman who experienced three episodes of generalized tonic-clonic seizures while exercising on a stair-climbing machine. [8] In that case, a cardiac stress test with simultaneous EEG demonstrated a generalized tonic-clonic seizure, and a sleep-deprived EEG showed two paroxysms of generalized discharges. As in our case, neuroimaging and interictal EEG were normal, and she had a normal cardiovascular response without arrhythmia or ST depression. [8] Even though the case illustrated the diagnostic utility of simultaneous cardiac stress testing with electroencephalography, it did not include a renal evaluation.

Three cases of generalized seizures triggered by jogging in healthy adults were described by Simpson et al. [9] In contrast to our patient, they presented findings on CT imaging that included small frontal lesions: an arteriovenous malformation, an astrocytoma, and a cyst. [9] No renal assessment was described for these cases, as a definitive diagnosis was reached through imaging. Ogunyemi et al. reported three cases, and Schmitt et al. described two patients with exercise-induced seizures. [6], [24] The five patients developed epilepsy early in childhood and later experienced seizures coinciding with physical activity. [6], [24] All of them had normal interictal EEGs with epileptic discharges during exercise. [6], [24] Brain imaging (CT/MRI) was normal in all cases, and a definitive etiology could not be determined. [6], [24] Even though Schmitt et al. reported performing extensive metabolic and endocrinologic investigations, no spot urinary electrolyte analysis was mentioned in these reports. [6], [24] Finally, Kamel et al. reported 10 cases of exercise-induced seizures, all of which were associated with abnormal temporal lobe activity detected by EEG, MRI, or PET. [25]

Although the specific mechanisms of exercise-induced seizures are uncertain, multiple precipitating factors have been described. These include fatigue, stress, repeated head trauma from contact sports, excessive aerobic activity, hyperventilation, altered metabolism of antiseizure medication, and ionic or metabolic disturbances. [1] However, no link has been established between post-exercise fatigue and increased seizure frequency; rather, it is described as beneficial for individuals with neurological diseases such as epilepsy. [1], [26] Scuba diving, skydiving, and other sports at heights are notable exceptions. [1] Our case points to an additional precaution that should be considered in patients with renal tubulopathies, where urinary electrolyte loss could induce brain imbalances that may trigger seizures.

In conclusion, we present a unique case of exercise-induced seizures secondary to a transient idiopathic renal tubulopathy marked by significant urinary loss of sodium, potassium, and chloride. This report highlights the importance of considering renal electrolyte disturbances, especially in the absence of neurological or cardiac abnormalities, in the diagnostic workup of exercise-related seizures. A spot urinary electrolyte analysis should be included when evaluating similar cases with otherwise normal imaging and interictal findings.

## 4 Consent

Written informed consent was obtained from the patient for publication of this case report and accompanying images. Reporting of this clinical case was approved by the IRB of the Instituto Oncológico Fundación Arturo López Pérez; Protocol ID: RPC14 | 2025-SN14-ALT-NML-PUB.

## Author contributions

All authors made a significant contribution to the work reported, whether that is in the conception, study design, execution, acquisition of data, analysis and interpretation, or in all these areas; took part in drafting, revising or critically reviewing the article; gave final approval of the version to be published; have agreed on the journal to which the article has been submitted; and agree to be accountable for all aspects of the work.

## CRediT authorship contribution statement

**Héctor M. Ramos-Zaldívar:** Writing – review & editing, Writing – original draft, Supervision, Methodology, Investigation, Formal analysis, Data curation, Conceptualization. **Eduardo Smelin Perdomo Domínguez:** Writing – review & editing, Writing – original draft, Investigation, Formal analysis, Data curation. **Joselin Michelle Monterroso-Reyes:** Writing – review & editing, Writing – original draft, Investigation, Formal analysis, Data curation. **Astrid Yohaly Rivera Caballero:** Writing – review & editing, Writing – original draft, Investigation, Formal analysis, Data curation. **Julie R. Jones:** Writing – review & editing, Writing – original draft, Investigation, Formal analysis. **Edison Salas-Huenuleo:** Writing – review & editing, Writing – original draft, Methodology, Investigation, Formal analysis. **María Fernanda Hernández Villarroel:** Writing – review & editing, Methodology, Investigation, Formal analysis. **Franz Villarroel-Espíndola:** Writing – review & editing, Methodology, Investigation. **Sashenka Silva-Jiménez:** Writing – review & editing, Writing – original draft, Investigation, Formal analysis. **Rodrigo E. Santa Cruz-Flores:** Writing – review & editing, Investigation, Formal analysis. **Cristobal Andrés Padilla Jeria:** Writing – review & editing, Investigation, Formal analysis. **Catalina Raquel González Urriola:** Writing – review & editing, Investigation, Formal analysis. **Martín P. Fierro:** Writing – review & editing, Investigation, Formal analysis.

## Funding

None.

## Declaration of competing interest

The authors declare that they have no known competing financial interests or personal relationships that could have appeared to influence the work reported in this paper.
